# First-principles investigation on elastic and thermodynamic properties of *Pnnm*-CN under high pressure

**DOI:** 10.1063/1.4972775

**Published:** 2016-12-16

**Authors:** Zhao Ya-Ru, Zhang Hai-Rong, Zhang Gang-Tai, Wei Qun, Yuan Yu-Quan

**Affiliations:** 1 College of Physics & Optoelectronics Technology, Baoji University of Arts and Sciences, Baoji 721016, China; 2 School of Electrical and Electronic Engineering, Baoji University of Arts and Sciences, Baoji 721016, China; 3 School of Sciences, Xidian University, Xi’an 710071, China; 4 School of Physics and Electronic Engineering, Sichuan University of Science & Engineering, Zigong 643000, China

## Abstract

The elastic anisotropy and thermodynamic properties of the recently synthesized *Pnnm*-CN have been investigated using first-principles calculations under high temperature and high pressure. The calculated equilibrium crystal parameters and normalized volume dependence of the resulting pressure agree with available experimental and theoretical results. Within the considered pressure range of 0–90 GPa, the dependences of the bulk modulus, Young’s modulus, and shear modulus on the crystal orientation for *Pnnm*-CN have been systematically studied. The results show that the *Pnnm*-CN exhibits a well-pronounced elastic anisotropy. The incompressibility is largest along the *c*-axis. For tension or compression loading, the *Pnnm*-CN is stiffest along [001] and the most obedient along [100] direction. On the basis of the quasi-harmonic Debye model, we have explored the Debye temperature, heat capacity, thermal expansion coefficient, and Grüneisen parameters within the pressure range of 0–90 GPa and temperature range of 0–1600K.

## I. INTRODUCTION

In material science, the superhard materials have attracted considerable interest owing to their far-ranging applications in cutting, polishing tools, and wear-resistant coatings.^1^ Because the compounds consisting of light elements display relatively short and strong covalent bonds, they usually possess low-compressibility and high hardness. Therefore, scientists made a great effort to synthesize or theoretically predict new covalent compounds. In 1989, Liu and Cohen^2^ suggested a new compound *β*-C_3_N_4_. which to be ultra-incompressible with a considerable bulk modulus (427 GPa) as that (442 GPa) of diamond.^3^ Subsequently, numerous studies searching for new CN compounds were carried out.^4–11^ Experiments selected different carbon-nitrogen rich compounds as precursors to synthesize CN phases, such as cyanamide, melamine, and other related triazine-based compounds.^12–14^ However, it is a faced challenge to determine the crystal structures, chemical compositions and internal atomic arrangement. To solve this problem, several theoretical approaches were employed to predict new CN materials.

One of the typical CN phase is the predicted *Pnnm-*CN, which has been successful synthesized by recent experiment. In 2012, Wang^15^ predicted an orthorhombic *Pnnm-*CN as the energetically most stable structure for carbon mononitride below 100 GPa. The theoretical results shown that the *Pnnm-*CN possesses the highest hardness (62.3 GPa) and can be synthesized using graphite and nitrogen as precursors at pressure of 10.9 GPa. Further interest is the experimental synthesis of *Pnnm-*CN under the condition of 55 GPa and 7000 K, as reported by Stavrou *et al.*.^16^ In their work, the pressure dependence of the lattice parameters of *Pnnm*-CN was discussed. Moreover, they repoted that the anisotropic behavior of *Pnnm-*CN was the high compressibility of *a*-axis. More recently, Tang *et al.* examined its mechanical properties by simulating the strain-stress relations at large strains. They pointed out that the weakest peak tensile stress of 41 GPa in the <100> direction and strongest peak tensile stress of 94 GPa in the <001> direction for *Pnnm*-CN.^17^ From above discussion, it is remarkable that the *Pnnm*-CN shows anisotropic behavior. Furthermore, few systematic studies on the elastic anisotropy and thermodynamic properties of the *Pnnm*-CN have been carried out until now.

In present work, the elastic properties of *Pnnm*-CN under pressure up to 90 GPa are studied, from which the elastic anisotropy is also found. Then the quasi-harmonic Debye model have been employed to explore the thermodynamic properties of the *Pnnm*-CN.

## II. COMPUTATIONAL METHODS

All first-principles calculations have been performed with the VASP package^18^ using the Perdew-Burke-Ernzrehof (PBE) generalized gradient approximation (GGA).^19^ The all electron projector augmented wave (PAW) method is adopted valence electrons of 2*s*^2^*p*^2^ and 2*s*^2^*p*^3^ for C and N atoms, respectively. The calculations of total energy and stress selected the energy cutoff of 800 eV and appropriate Monkhorst-Pack *k* meshes^20^ of 8×10×16. The elastic constants of the *Pnnm*-CN under different pressure have been obtained via strain-stress approach. In the light of the Voigt-Reuss-Hill approximation,^21^ one can calculate the bulk modulus, shear modulus, Young’s modulus, and Poisson’s ratio. Furthermore, the thermodynamics properties of the *Pnnm*-CN are also investigated according to the quasi-harmonic Debye model.

## III. RESULTS AND DISCUSSION

### A. Structural properties

**FIG. 1.**
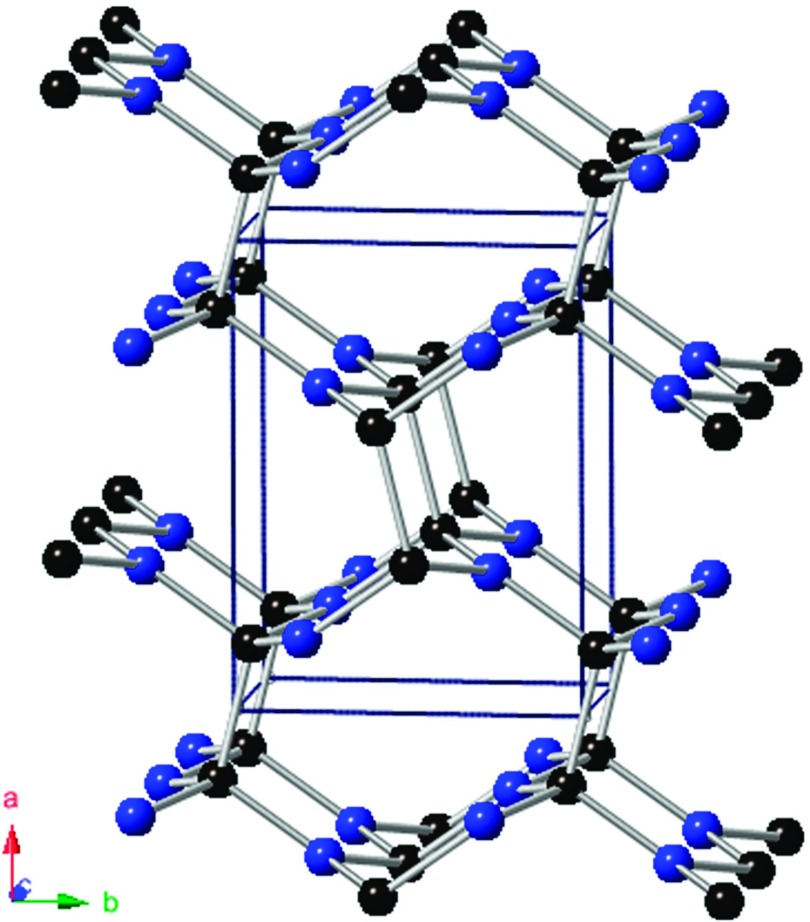
Crystal structure of *Pnnm*-CN. The black and blue spheres represent C and N atoms, respectively.

**FIG. 2.**
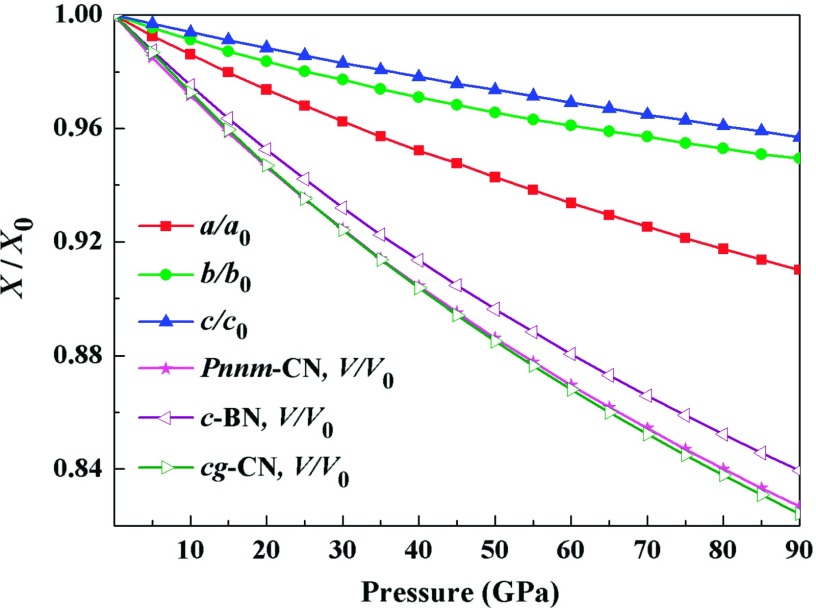
The normalized parameters *a/a*_0_, *b/b*_0_, *c/c*_0_, and *V/V*_*0*_ as a function of pressure for *Pnnm*-CN.

The crystal structure of the *Pnnm*-CN is displayed in Fig. 1. In Table I, we list our calculated lattice parameters as well as the previous experimental and theoretical data. It is clear that our results are completely closed to the theoretical values at 0 GPa and 10 GPa.^15,17^ The mismatch of the lattice parameters at 55 GPa is within 6% in comparison with recent experimental data.^16^ Furthermore, the relationships of the normalized parameters $a/a_{0}$, $b/b_{0}$, $c/c_{0}$, and $V/V_{0}$ against pressure are shown in Fig. 2, where *a*_0_, *b*_0_, *c*_0_, and *V*_*0*_ are the equilibrium structural parameters at 0 GPa and 0 K. The fitting relationships at 0 K are to found:

$$\frac{a}{a_{0}}=0.99904-1.32\times 10^{-3}P+3.70317\times 10^{-6}P^{2}$$ (1)

$$\frac{b}{b_{0}}=0.99942-0.83596\times 10^{-3}P+3.17792\times 10^{-6}P^{2}$$ (2)

$$\frac{c}{c_{0}}=0.99982-0.592175\times 10^{-3}P+1.32158\times 10^{-6}P^{2}$$ (3)

**TABLE I.** The theoretical and experimental structural parameters of *Pnnm*-CN at 0 GPa, 10 GPa, and 55 GPa.

Pressure	Source	*a*	*b*	*c*	*V*_0_
0 GPa	This work	5.333	3.950	2.374	6.251
	Theoretical^17^	5.335	3.952	2.374	6.257
	Theoretical^15^				6.12
10 GPa	This work	5.259	3.916	2.360	6.075
	Theoretical^15^	5.2579	3.9181	2.3602	6.078
55 GPa	This work	5.003	3.805	2.304	5.482
	Exprimental^16^	4.77	3.67	2.45	

One can see that the incompressibility is the largest along the *c*-axis, whereas it is smallest along *a*-axis. The clear elastic anisotropy of *Pnnm*-CN is displayed. The low incompressibility of *a*-axis might originate from the tilting of the C-C dumbbells with respect to *a*-axis. In addition, we notice that the incompressibility of volume for *Pnnm*-CN is better than that of *cg*-CN at high pressure, although it is lower than that of *c*-BN.

### B. Elastic properties

**FIG. 3.**
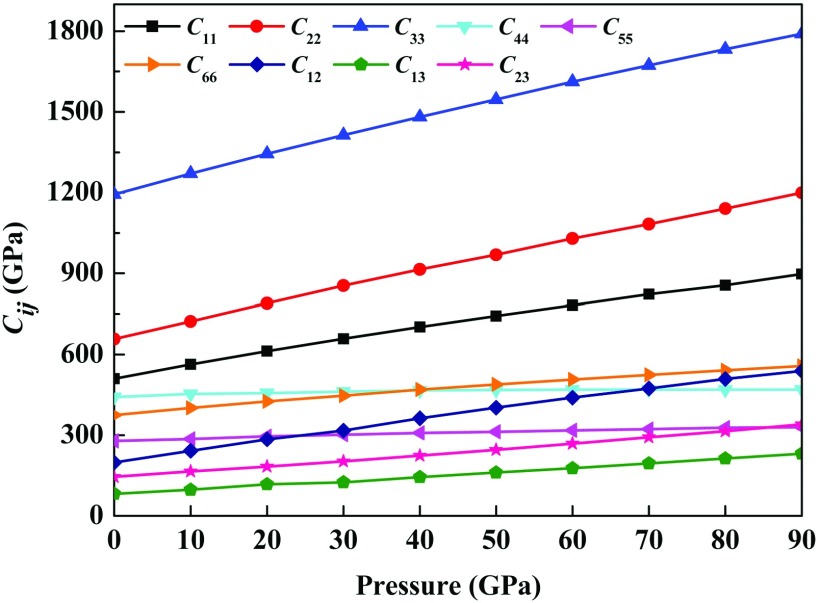
The calculated elastic constants as a function of pressure for *Pnnm*-CN.

By strain-stress method, the elastic constants *C*_*ij*_ of *Pnnm*-CN are calculated and listed in Table II. From the table, one can find that the present data accord with the values reported in Refs. 10 and 17 at 0 GPa and 0 K. In Fig. 3, the variations of elastic constants with pressure up to 90 GPa are plotted. It is found that the value of *C*_*ij*_ against the applied pressure *P* increase monotonically. Relatively, the values of *C*_33_ increase sharply against the pressure growing from 0 to 90 GPa, while those of *C*_44_ and *C*_55_ are slower. Up to 90 GPa, the *C*_*ij*_ still satisfy the condition of the Born-Huang criteria,^22^ illustrating that the *Pnnm*-CN is mechanical stable at high pressure. This is consistent with the result reported by Dong *et al.*^23^ According to the Voigt-Reuss-Hill approximation, we can obtain the bulk modulus *B* and shear modulus *G*, as listed in Table II. As increasing pressure, it is seen that both *B* and *G* show a monotonic growth. Pugh^24^ suggested that the brittle/ductile characteristics of materials can be estimated by the ratio of *B*/*G*. A material is defined to be ductile if B/G>1.75, otherwise it is brittle. For the *Pnnm*-CN, the ratio of *B*/*G* at 0 GPa is 1.04, which suggests that it is a brittle material. When the pressure increases from 0 GPa to 90 GPa, the value of *B*/*G* increases from 1.04 to 1.54. It indicates that the *Pnnm*-CN can become less brittle under high pressure. This results would be further confirmed by the Poisson’s ratio. Frantsevich *et al.*,^25^ suggested that the Poisson’s ratio ν of the brittle materials should be lower than 1/3. From the Table II, the lower values (0.139–0.242) of ν illustrates that the *Pnnm*-CN is a brittle material at the pressure range of 0–90 GPa.

**TABLE II.** Calculated elastic constants *C*_*ij*_ (GPa), bulk modulus *B* (GPa), shear modulus *G* (GPa), Young’s modulus *E* (GPa), Poisson’s ratio ν, and *G/B* of *Pnnm*-CN under pressure (GPa).

P	*C*_11_	*C*_22_	*C*_33_	*C*_44_	*C*_55_	*C*_66_	*C*_12_	*C*_13_	*C*_23_	B	G	E	G/B	ν
0^a^	506	643	1183	442	275	372	191	80	140	336	326		1.03	
0^b^	518	767	1127	534	277	379	203	83	201	369	351	799	1.05	0.139
0	510	657	1193	442	278	374	198	82	145	342	328	746	1.04	0.137
10	563	722	1271	453	286	401	242	97	165	382	344	794	1.11	0.154
20	612	789	1344	457	296	426	285	117	184	422	358	837	1.18	0.169
30	658	854	1414	462	302	447	317	125	203	455	372	877	1.22	0.179
40	702	914	1481	466	308	469	363	144	224	493	382	911	1.28	0.192
50	743	969	1546	468	313	489	402	161	245	528	392	943	1.35	0.202
60	782	1029	1612	469	318	507	439	177	269	562	401	973	1.41	0.212
70	822	1083	1673	469	322	524	474	195	292	596	410	1001	1.45	0.220
80	856	1140	1732	469	327	541	509	213	315	629	418	1028	1.49	0.228
90	897	1199	1790	469	330	557	539	232	340	662	428	1056	1.54	0.234

^a^ Ref. 17.

^b^ Ref. 15.

In the crystal physics and engineering science, the elastic anisotropy is an important index for materials. Elastic anisotropy can provide an expectation of the atoms arrange, the bonding properties, and some chemical characters in different directions of materials.^26^ It is well known that the shear anisotropic factor can reflect the level of anisotropy for different planes. For the (100) shear plane between the [011] and [010] directions, the shear anisotropic factor A_1_ can be written as the following formula:^27^

$${\text{A}}_{1}=\frac{4C_{44}}{C_{11}+C_{33}-2C_{13}}.$$ (4)

For the (010) shear planes between [101] and [001] directions, A_2_ is^28^

$${\text{A}}_{2}=\frac{4C_{55}}{C_{22}+C_{33}-2C_{23}}.$$ (5)

For the (001) shear planes between [110] and [010] directions, A_3_ is^28^

$${\text{A}}_{3}=\frac{4C_{66}}{C_{11}+C_{22}-2C_{12}}.$$ (6)

**FIG. 4.**
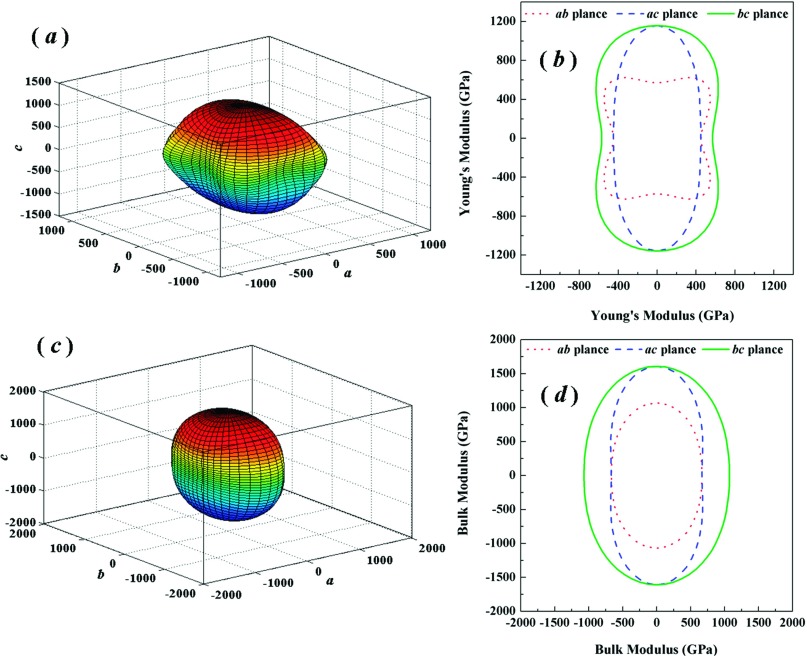
Three-dimensional surface depictions (*a*) and plane projections of the directional dependence of the Young’s modulus (*b*); Three-dimensional surface depictions (*c*) and plane projections of the directional dependence of the bulk modulus (*d*).

For an isotropic materials, the shear anisotropy factors must be 1.0. Any departure from 1.0 can reflect the level of elastic anisotropy. From the Table III, it is interesting to note that the anisotropy of the (001) shear planes between [110] and [010] directions is the largest and (100) shear plane between the [011] and [010] directions is smallest. Although A_3_ increases and A_2_ decrease with increasing pressure, the anisotropy of both (001) shear planes between [110] and [010] directions and the (010) shear planes between [101] and [001] directions changes more distinct. Obviously, the shear anisotropy factors are insufficient to reflect the anisotropy behavior of crystal completely. A straightforward way is the three-dimensional (3D) surface depictions, which can display the variation of the elastic modulus with the crystallographic direction. For orthorhombic phase, the dependence of the Young’s modulus *E* and bulk modulus *B* on crystallographic direction are expressed by:^27^

$$\begin{array}{cc} E^{-1} & =S_{11}\alpha^{4}+S_{22}\beta^{4}+S_{33}\gamma^{4}+2S_{12}\alpha^{2}\beta^{2}+2S_{23}\beta^{2}\gamma^{2}+2S_{13}\alpha^{2}\gamma^{2}\\ & +S_{44}\beta^{2}\gamma^{2}+S_{55}\alpha^{2}\gamma^{2}+S_{66}\alpha^{2}\beta^{2}, \end{array}$$ (7)

$$B^{-1}=\left(S_{11}+S_{12}+S_{13}\right)\alpha^{2}+\left(S_{12}+S_{22}+S_{23}\right)\beta^{2}+\left(S_{13}+S_{23}+S_{33}\right)\gamma^{2},$$ (8)

in which *S*_*ij*_ represent the elastic compliance constants given by Nye.^29^ The α, β, and γ represent the direction cosines of [*uvw*] direction. The shear modulus *G* on the (*hkl*) shear plane with the shear stress applied alone the [*uvw*] direction is written as^30^

$$\begin{array}{cc} G^{-1}= & 4S_{11}\alpha_{1}^{2}\alpha_{2}^{2}+4S_{22}\beta_{1}^{2}\beta_{2}^{2}+4S_{33}\gamma_{1}^{2}\gamma_{2}^{2}+8S_{12}\alpha_{1}\alpha_{2}\beta_{1}\beta_{2}\\ & +8S_{23}\beta_{1}\beta_{2}\gamma_{1}\gamma_{2}+8S_{13}\alpha_{1}\alpha_{2}\gamma_{1}\gamma_{2}+S_{44}{\left(\beta_{1}\gamma_{2}+\beta_{2}\gamma_{1}\right)}^{2}\\ & +S_{55}{\left(\alpha_{1}\gamma_{2}+\alpha_{2}\gamma_{1}\right)}^{2}]+S_{66}{\left(\alpha_{1}\beta_{2}+\alpha_{2}\beta_{1}\right)}^{2}, \end{array}$$ (9)

where $\alpha_{1}$, $\beta_{1}$, $\gamma_{1}$, $\alpha_{2}$, $\beta_{2}$, $\gamma_{2}$ are the direction cosines of the [*uvw*] and [*HKL*] directions in the coordinate systems, and the [*HKL*] direction shows the vector normal to the (*hkl*) shear plane.^30^ Figs. 4(a) and (c) display the 3D surface depictions of the *E* and *B*. For an isotropic crystal, the 3D surface depictions should be the spheric shape. A divergence from the spheric shape may well reflect the level of the elastic anisotropy. For the *Pnnm*-CN, both *E* and *B* show large divergence from the spheric shape. Hence, It is concluded that the *Pnnm*-CN exhibits a significant elastic anisotropy. In addition, the projections of 3D surface depictions of both *E* and *B* on the *ab*, *ac*, and *bc* planes have also been shown in Figs. 4(b) and (d). It is clear that the *Pnnm*-CN exhibits in-plane anisotropy in all planes, especially in *ac* plane. To further understand the anisotropy of the Young’s modulus *E* along different directions, the dependence of the *E* on orientation is studied when we take the tensile axis within given plane. For the (001) plane, let θ be the angle of between [100] and [*uv*0]. The equation (7) can be deduced as:^26^

$$E^{-1}=s_{11}\cos^{4}\theta +s_{22}\sin^{4}\theta +2s_{12}\sin^{2}\theta \cos^{2}\theta +s_{66}\sin^{2}\theta \cos^{2}\theta .$$ (10)

For the (100) plane, let *θ* be the angle of between [001] and [0vw]. The equation (7) can be deduced as:^26^

$$E^{-1}=s_{22}\sin^{4}\theta +s_{33}\cos^{4}\theta +\frac{1}{4}\left(2s_{23}+s_{44}\right)\sin^{2}2\theta .$$ (11)

For the (010) plane, let θ be the angle of between [001] and [*u*0*w*]. The equation (7) can be deduced as:^26^

$$E^{-1}=s_{11}\sin^{4}\theta +s_{33}\cos^{4}\theta +\frac{1}{4}\left(2s_{13}+s_{55}\right)\sin^{2}2\theta .$$ (12)

**FIG. 5.**
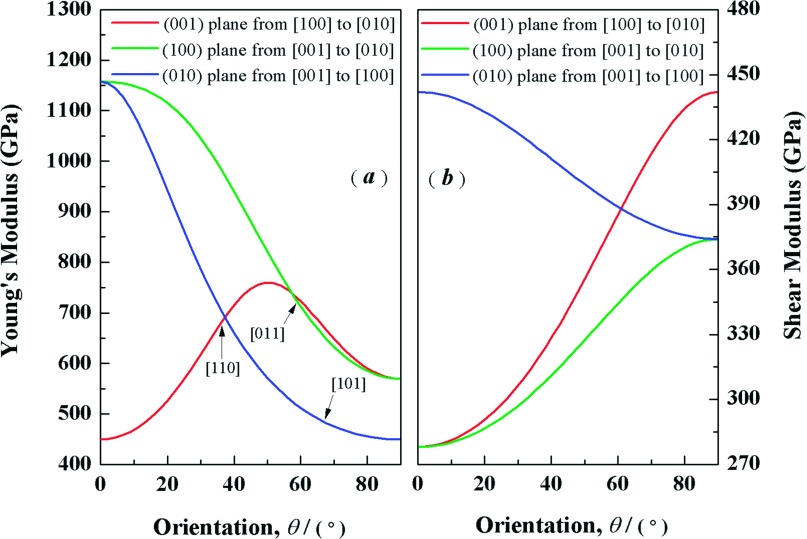
Orientation dependences of Young’s modulus (*a*) and shear modulus (*b*) for *Pnnm*-CN.

The orientation dependence of the *E* are plotted in Fig. 5(a). As shown in the figure, some interesting results can be obtained. First, the value of *E* is dependent on the direction of the tensile stress. Second, *Pnnm*-CN has a maximum of E=758GPa and a minimum of $E_{\left[100\right]}=449\text{GPa}$ from [100] to [010] in the (001) plane. For the (100) plane, the value of *E* alone the [001] direction is the maximal value (1157 GPa) and the [010] direction is the minimal one (570 GPa). From [001] to [100] directions in the (010) plane, it shows a maximum of $E_{\left[001\right]}=1157\text{GPa}$ and a minimum of $E_{\left[100\right]}=449\text{GPa}$. Third, we can obtain the values of *E* alone the [110], [011], and [101] directions in (001), (100), and (010) planes, respectively. The results are $E_{\left[110\right]}=681$ GPa, $E_{\left[011\right]}=719$ GPa, and $E_{\left[101\right]}=485$ GPa. Finally, the ordering of the Young’s modulus at different directions for the *Pnnm*-CN is: $E_{\left[001\right]}>E_{\left[011\right]}>E_{\left[110\right]}>E_{\left[010\right]}>E_{\left[101\right]}>E_{\left[100\right]}$.

**TABLE III.** Calculated anisotropy factor of the *Pnnm*-CN under pressure.

P (GPa)	*A*_1_	*A*_2_	*A*_3_
0	1.149	0.713	1.940
10	1.105	0.688	2.002
20	1.062	0.671	2.051
30	1.014	0.649	2.036
40	0.984	0.633	2.108
50	0.952	0.618	2.154
60	0.920	0.605	2.174
70	0.891	0.593	2.190
80	0.868	0.583	2.213
90	0.844	0.572	2.189

To investigate the plastic deformation of *Pnnm*-CN, the variation of the shear modulus *G* with the shear stress direction has been investigated and plotted in Fig. 5(b). For the (001) plane, we rotate the stress direction from [100] to [010]. The Eq. (9) can be deduced as: $G^{-1}=s_{55}+\left(s_{44}-s_{55}\right)\sin^{2}\theta$. As for *Pnnm*-CN, we can obtain the smallest value of shear modulus $G_{\min}=278\text{GPa}$ along [100] and largest value $G_{\max}=442\text{GPa}$ along [010], respectively. When we take the (100) plane and change the orientation from [001] to [010], the Eq. (9) can be deduced as: $G^{-1}=s_{55}+\left(s_{66}-s_{55}\right)\sin^{2}\theta$. In the (100) plane, the value of *G* is the smallest alone the [001] direction ($G_{\max}=278\text{GPa}$) and the largest along the [010] direction ($G_{\min}=442\text{GPa}$). If the shear plane is (010) with varied orientation from [001] to [100], $G^{-1}=s_{44}+\left(s_{66}-s_{44}\right)\sin^{2}\theta$. The value of *G* has maximum of 442 GPa along [001] and minimum of 374 GPa along [100]. Through the above analysis, it is remarkable that the *Pnnm*-CN exhibits a significant elastic anisotropy.

### C. Thermodynamics properties

The calculations on thermodynamics properties as functions of temperature and pressure are necessary for solids in terms of quasi-harmonic Debye model.^31^ It is worth mentioning that this model was successfully used to predict the thermodynamic properties of some materials.^32–36^ According to the model, the thermodynamics properties of the *Pnnm*-CN have been discussed in the following. In Table IV, we list the results of Debye temperature *Θ* at 0, 10, 20, 30, 40, 50, 60, 70, 80, and 90 GPa and 0, 200, 400, 600, 800, 1000, 1200, 1400, and 1600 K. For the given temperature, *Θ* increases sharply with increasing pressure. When the pressure keeps constant, *Θ* reduces gradually with the growth of the temperature, displaying the nearly linear relationship within the pressure range of 40–90 GPa. When the applied pressure increases from 0 to 90 GPa, the values of *Θ* increase by 40.06%, 40.15%, 40.93%, 42.43%, 44.45%, 45.87%, 49.9%, 53.62%, and 58.37% at 0, 200, 400, 600, 800, 1000, 1200, 1400, and 1600 K, respectively. When the applied *T* increases from 0 to 1600 K, they reduce by 13.49%, 9.39%, 7.13%, 5.69%, 4.68%, 3.92%, 3.34%, 2.88%, 2.51%, and 2.19% at 0, 10, 20, 30, 40, 50, 60, 70, 80, and 90 GPa, respectively. It is clearly that the effects of pressure on the *Θ* are significant than those of temperature on it.

**TABLE IV.** The calculated Debye temperature (Θ/K) of the *Pnnm*-CN under different pressures and temperatures.

	*P* (GPa)									
*T* (K)	0	10	20	30	40	50	60	70	80	90
0	1597.3	1700.6	1791.4	1872.7	1946.4	2013.9	2076.1	2133.7	2187.3	2237.2
200	1596.2	1699.8	1790.9	1872.3	1946.1	2013.6	2075.9	2133.5	2187.1	2237.1
400	1586.2	1692.5	1785.1	1867.6	1942.2	2010.4	2073.2	2131.2	2185.2	2235.4
600	1566.4	1677.2	1772.6	1857.3	1933.6	2003.0	2066.7	2125.6	2180.2	2231.0
800	1540.1	1656.5	1755.8	1843.1	1921.4	1992.4	2057.5	2117.4	2173.0	2224.6
1000	1519.4	1632.2	1734.6	1825.8	1907.2	1980.4	2047.0	2107.9	2164.2	2216.4
1200	1472.9	1605.0	1714.0	1808.0	1891.1	1965.9	2034.1	2096.6	2154.4	2207.9
1400	1431.1	1574.7	1689.8	1787.8	1873.8	1950.9	2020.8	2084.9	2143.9	2198.4
1600	1381.8	1540.9	1663.6	1766.2	1855.4	1935.0	2006.8	2072.2	2132.5	2188.3

**FIG. 6.**
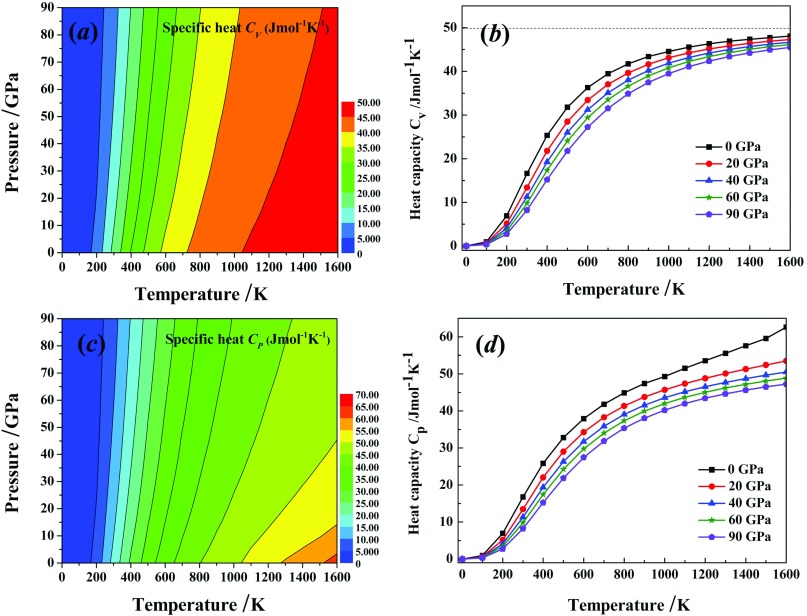
The calculated specific heat capacity at constant volume *C*_*V*_ and at constant pressure *C*_*P*_ as a function temperature for *Pnnm*-CN at different pressures: *C*_*V*_ contours (*a*), $C_{V}-T$ (*b*), *C*_*P*_ contours (*c*), $C_{P}-T$ (*d*).

As one of the most significant thermodynamics parameters of the solids, the heat capacity not only provides available information of the vibrational properties but also is fundamental for many applications.^37^ The temperature dependence of the heat capacity at various pressures for *Pnnm*-CN is displayed in Fig. 6. It is clear that both heat capacity at constant volume (*C*_*V*_) and heat capacity at constant pressure (*C*_*P*_) increase with temperature at the same pressure, while decrease with pressure at the same temperature. In more detail, both *C*_*V*_ and *C*_*P*_ of the *Pnnm*-CN follow the law of *T*^3^ at low temperature. At high temperature, *C*_*P*_ increases persistently, while *C*_*V*_ increases slightly and closes to a constant of 3*Nk*_*B*_ (≈49.9Jmol^-1^K^-1^) at sufficient high temperature. The difference between *C*_*V*_ and *C*_*P*_ can be expressed as $C_{P}=C_{V}+TV\alpha^{2}$.^38^ At low temperature, the value of *α* mainly lead to the departure of *C*_*V*_ and *C*_*P*_. In case like this, there is small departure of *C*_*V*_ and *C*_*P*_ duo to small value of *α*. At high temperature, the behavior of the *C*_*V*_ is obeying the law of Dulong-Petit, while the value of *C*_*P*_ is proportional to *T*. Therefore, the departure of *C*_*V*_ and *C*_*P*_ is obvious. In addition, the dependence of both *C*_*V*_ and *C*_*P*_ on *T* are greater than that on the *P*.

**FIG. 7.**
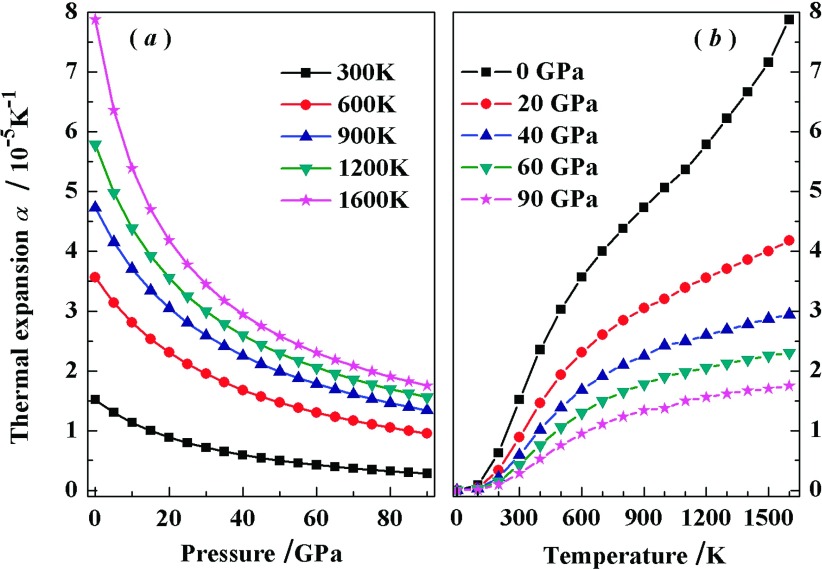
Variation of the thermal expansion coefficient with pressure for *Pnnm*-CN at different temperatures (*a*). Variation of the thermal expansion coefficient with temperature for *Pnnm*-CN at different pressures (*b*).

Thermal expansion coefficient α reflects the change of solid volume in response to the change in pressure *P* or temperature *T*.^33^ The variations of α on *P* and *T* are illustrated in Fig. 7. It is shown that the α, under certain temperature, decreases sharply at P≤40GPa, then changes slowly at P>40GPa. There are small influence of pressure on α at low temperature. As shown in Fig. 7(b), the α increases quickly with *T* especially for low temperature and 0 GPa, then it reaches to a linear increase under high temperatures. It is explained by the relation of $\alpha ∼C_{V}/B$.^31^ The bulk modulus slowly and linearly reduces with temperature. At low temperature, the quick increase of *C*_*V*_ mainly cause the remarkable variation of α. At high temperature, the α shows a linear increase depended on *B* because the *C*_*V*_ nearly approaches to Dulong-Petit limit.

**FIG. 8.**
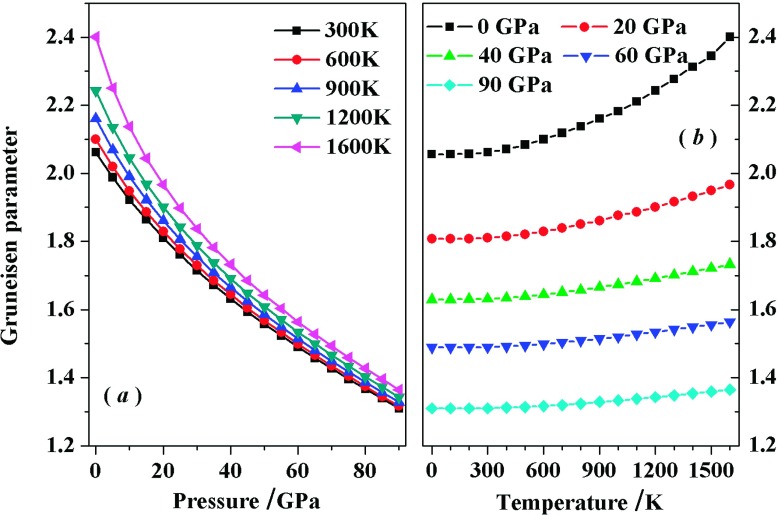
Variation of the Grüneisen parameter with pressure for *Pnnm*-CN at different temperatures (*a*). Variation of the thermal expansion coefficient with temperature for *Pnnm*-CN at different pressures (*b*).

As a key thermodynamic quantities: the Grüneisen parameter γ reflects the anharmonic effects in the vibrating lattice.^39^ In Fig. 8, we have displayed the Grüneisen parameter γ of *Pnnm*-CN at various temperatures and pressures. For the given temperature, γ decreases sharply with *P*, especially at high temperature. Meanwhile, the variations of γ with *P* almost display a linear relationship in the pressure range of 40–90 GPa. For the given pressure, γ increases obviously with increasing temperature at P≤40GPa, then increases monotonously with increasing temperature at P>40GPa. The influences of *P* on γ are greater than *T*.

## IV. CONCLUSIONS

At high temperature and high pressure, the elastic anisotropy and thermodynamic properties of the recently synthesized *Pnnm*-CN have been systematically investigated. The calculated equilibrium crystal parameters and normalized volume at given pressure are completely closed to previous experimental and theoretical data. To understand the elastic anisotropy of *Pnnm*-CN, the relationships of the Young’s modulus and shear modulus against crystal orientation for *Pnnm*-CN are discussed. The evidence of the obvious elastic anisotropy for *Pnnm*-CN is obtained. Using quasi-harmonic Debye model, the thermodynamic properties, such as the Debye temperature, heat capacity, thermal expansion coefficient, and Grüneisen parameter, of *Pnnm*-CN have also been investigated under high pressure and high temperature.
